# Assessment formats in dental medicine: An overview

**DOI:** 10.3205/zma001064

**Published:** 2016-08-15

**Authors:** Susanne Gerhard-Szep, Arndt Güntsch, Peter Pospiech, Andreas Söhnel, Petra Scheutzel, Torsten Wassmann, Tugba Zahn

**Affiliations:** 1 Goethe-Universität, Carolinum Zahnärztliches Universitäts-Institut gGmbH, Poliklinik Zahnerhaltungskunde, Frankfurt am Main, Deutschland; 2 Marquette University School of Dentistry, Department of Surgical Sciences, Milwaukee, USA und Universitätsklinikum Jena, Zentrum für Zahn-, Mund- und Kieferheilkunde, Jena, Deutschland; 3 Universität Würzburg, Poliklinik für Zahnärztliche Prothetik, Würzburg, Deutschland; 4 Universitätsmedizin Greifswald, Poliklinik für Zahnärztliche Prothetik, Alterszahnheilkunde und medizinischer Werkstoffkunde, Greifswald, Deutschland; 5 Universitätsklinikum Münster, Poliklinik für Prothetische Zahnmedizin & Biomaterialien, Münster, Deutschland; 6 Universitätsmedizin Göttingen, Poliklinik für Zahnärztliche Prothetik, Göttingen, Deutschland; 7 Goethe-Universität, Carolinum Zahnärztliches Universitäts-Institut gGmbH, Poliklinik für Zahnärztliche Prothetik, Frankfurt am Main, Deutschland

## Abstract

**Aim: **At the annual meeting of German dentists in Frankfurt am Main in 2013, the Working Group for the Advancement of Dental Education (AKWLZ) initiated an interdisciplinary working group to address assessments in dental education. This paper presents an overview of the current work being done by this working group, some of whose members are also actively involved in the German Association for Medical Education's (GMA) working group for dental education. The aim is to present a summary of the current state of research on this topic for all those who participate in the design, administration and evaluation of university-specific assessments in dentistry.

**Method**: Based on systematic literature research, the testing scenarios listed in the National Competency-based Catalogue of Learning Objectives (NKLZ) have been compiled and presented in tables according to assessment value.

**Results: **Different assessment scenarios are described briefly in table form addressing validity (V), reliability (R), acceptance (A), cost (C), feasibility (F), and the influence on teaching and learning (EI) as presented in the current literature. Infoboxes were deliberately chosen to allow readers quick access to the information and to facilitate comparisons between the various assessment formats. Following each description is a list summarizing the uses in dental and medical education.

**Conclusion: **This overview provides a summary of competency-based testing formats. It is meant to have a formative effect on dental and medical schools and provide support for developing workplace-based strategies in dental education for learning, teaching and testing in the future.

## Zusammenfassung

**Ziele:** Auf Initiative des Arbeitskreises für die Weiterentwicklung der Lehre in der Zahnmedizin (AKWLZ) wurde 2013 auf dem Deutschen Zahnärztetag in Frankfurt am Main eine interdisziplinäre Arbeitsgruppe zum Thema „Prüfungen in der Zahnmedizin“ gegründet. Diese Übersicht stellt die Zusammenfassung der aktuellen Arbeit dieser AG dar, deren Mitglieder zum Teil auch in der Arbeitsgruppe Zahnmedizin der Gesellschaft für Medizinische Ausbildung (GMA) aktiv mitwirken. Ziel der vorliegenden Übersichtsarbeit ist es, allen Interessierten, die sich mit der Planung, Durchführung und Auswertung von fakultätsinternen Prüfungen im Fach Zahnmedizin beschäftigen, den aktuellen Forschungsstand zum Thema darzustellen und zusammenzufassen.

**Methoden: **Basierend auf einer systematischen Literaturrecherche wurde anlehnend am sogenannten Nutzwertindex von Prüfungen eine tabellarische Darstellung der im NKLZ aufgeführten Szenarien realisiert.

**Ergebnisse:** Unterschiedliche Prüfungsszenarien wurden nach einer kurzen Beschreibung bezüglich ihrer Validität / Gültigkeit (V), der Reliabilität / Zuverlässigkeit (R), der Akzeptanz (A), der Kosten (C), der Durchführbarkeit (F) und des Einflusses auf Lernen und Lehren (EI) nach aktuellem Stand der Literatur tabellarisch dargestellt. Die Darstellungsform der Infoboxen wurde bewusst gewählt, um den Interessierten einen schnellen Einstieg in und Vergleich der einzelnen Prüfungsszenarien zu ermöglichen. Am Ende jeder Prüfungsbeschreibung befinden sich eine stichwortartige Sammlung der jeweiligen Anwendung in der Zahn(Medizin) und ein Fazit für die Anwendung.

**Folgerungen: **Die vorliegende Übersichtsarbeit beinhaltet eine Zusammenfassung zum Thema von kompetenzbasierten Prüfungsszenarien. Sie soll einen formativen Effekt auf die (zahn)medizinischen Fakultäten haben und sie darin unterstützen, die für die Zahnmedizin typischen Schwerpunkte des arbeitsplatzbasierten Lernens, Lehrens und Prüfens in der Zukunft weiterzuentwickeln.

## 1. Starting point

A concerted connection between teaching and testing (constructive alignment) is crucial to impart dental competencies during university study [1].

**Figure 1:**
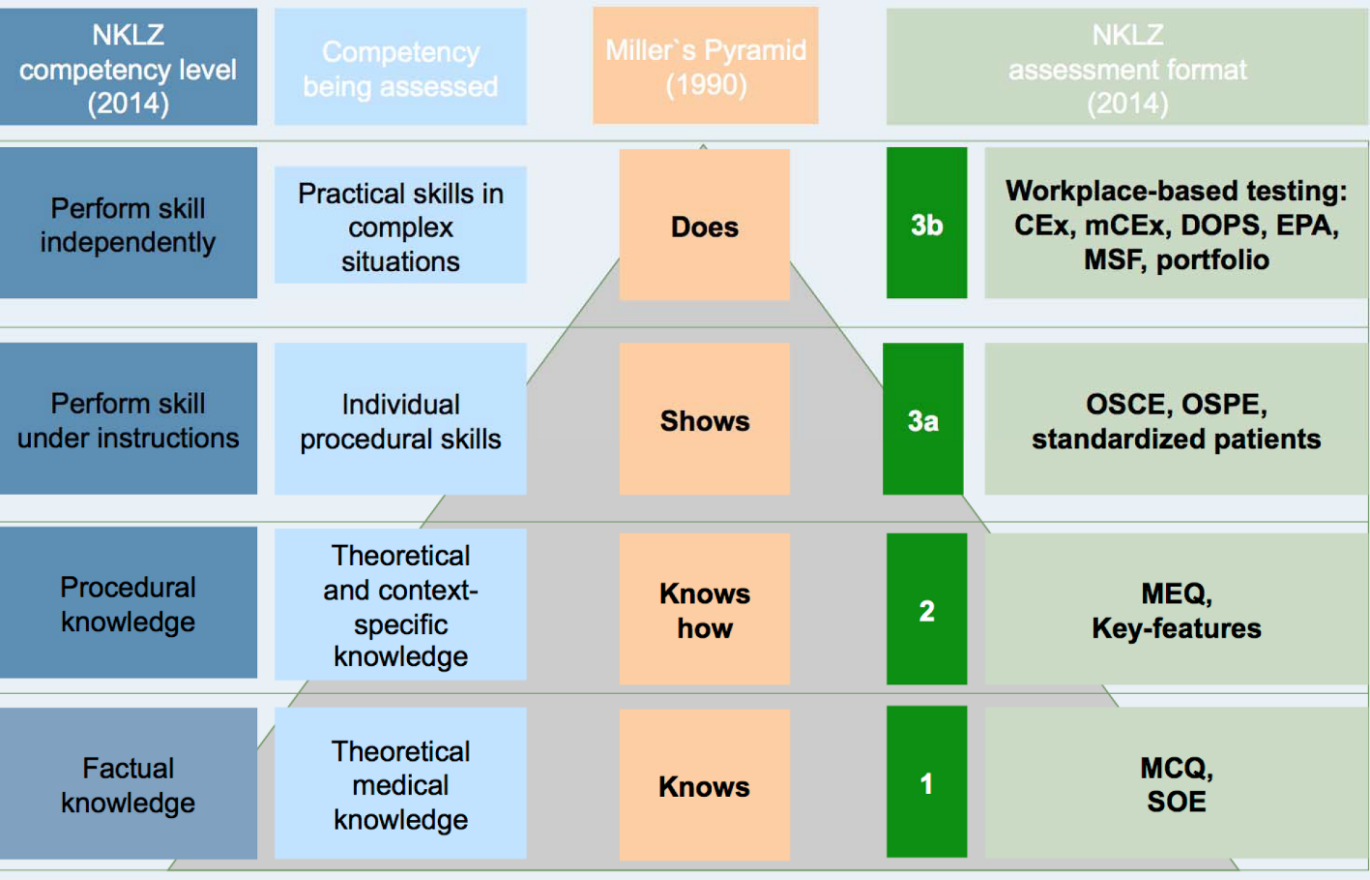
Examples of assessment scenarios depending on competency level according to the requirements in Miller and the National Competency-based Catalogue of Learning Objectives for Undergraduate Dental Education (NKLZ).

Also connected with the definition of competency-based learning objectives are the appropriate testing formats that measure the requisite combination of knowledge, practical skills and ability to engage in professional decision-making for each particular task (see Figure 1 (Fig. 1)). 

## 2. Method

A survey of the literature was undertaken between January 17 and December 17, 2014, in the databases of the German National Library (DNB), MEDLINE using the PubMed interface, Excerpta Medica Database (EMBASE), Education Resource Information Centre (ERIC), Cochrane Library, Science Citation Index, and Google Scholar. The search was conducted automatically and supplemented manually. In addition, available dissertations, open-access publications by German Medical Science (GMS), BEME (Best Evidence Medical and Health Professional Education), including German-language conference proceedings, such as the AKWLZ and GMA, were evaluated. The search terms included the German equivalents for “MCQ”; “MEQ”; “multiple choice”; “MC”; “multiple-choice questionnaire”; “SMP”; “structured oral examination”; “SOE”; “key feature”; “OSCE”; “OSPE”; “standardized patient”; “CEX”; “miniCEX”; “entrustable professional activities”; “DOPS”; “portfolio”, “multi-source feedback” in combination with “AND” and “dental”; “medicine”; “education”; “assessment.”

In an initial step, literature was selected based on title and abstract in accordance with pre-defined inclusion and exclusion criteria (inclusion criteria: published 1966-2013 in German or English with topical relevance; exclusion criteria: failure to meet inclusion criteria, full text not available in English or German, lack of topical relevance). The selected publications were then evaluated in terms of their relevance to the issue at hand and excluded, as required.

The articles were analyzed, and the results were described according to categories based on the value of assessments [2]. These categories cover the parameters of validity (V), reliability (R), acceptance (A), cost (C), feasibility (F) and influence on teaching and learning (EI). The results were then organized according to the evaluation parameters above. These criteria were further developed in 2011 by the working group headed by Norcini, and the parameters of “equivalence” (assessments conducted at different sites) and “catalytic effect” (consequences for the medical school) were added [3]. In this overview, both of these additional parameters were included and discussed in text form. The focus of this analysis has been carried out using the six criteria listed above (V, R, A, C, F, EI) in the form of tables to allow for clarity and comparisons.

## 3. Results

In total, n=223 publications were identified using the search strategy outlined above and drawn upon as the basis for the following analyses. 

## 4. Discussion

Structured oral examinations and the** multiple-choice questionnaire (MCQ**) are suited for testing theoretical knowledge, meaning descriptive knowledge of competency level 1 [4], [5], [6], [7]. The MCQ is a written assessment with several response options (closed questions), of which a single choice or multiple ones (multiple choice, multiple select) can be the right answer. After a brief introduction of the content and question come the response options that include the correct answer(s) and distracters (wrong answers). Multiple-choice exams can be paper-based, combined with computer-assisted grading, or even administered entirely at computer workstations [4].

Use in medical and dental education

- MCQs are presently used in both medical and dental study programs [6], [8].
- The most important preliminary and final examinations include multiple-choice questions: the preliminary exam in natural science (NVP), preliminary dental exam (ZVP), dental exam (ZP), first and second state medical exams (ÄP). Moreover, MCQs are specifically found in all pre-clinical and clinical subjects in both study programs; this type of question represents one of the most traditional, predominating assessment formats [4], [8].

To assess factual knowledge, the MCQ offers a cost-efficient testing format with high reliability and validity if the questions correspond to the quality criteria. With MCQs it is possible to objectively test a large amount of content in a short period of time. However, this type of assessment can lead to superficial learning of facts. 

### Multiple-choice Questionnaire

**Validity**

- Classified as high [9]
- Quality criteria for questions must be met to have sufficient validity [10].
- A high construct validity can be achieved if questions are subjected to a review process (e.g. via Item Management System [IMS]) [11].

**Reliability**

- Classified as high [9], [11]
- A minimum of 40 high-quality questions are needed to yield α Cronbach’s α of 0.8 [6].

**Acceptance**

- Scoring is objective [4].
- MCQs are considered fair if what has been taught corresponds with what is tested [12].
- The possibility of passing by giving “strategic” responses, guessing, or picking up on cues is viewed critically by teachers [13], [14], [15], [16].

**Cost**

- In light of the numbers and frequency of tests, it is an effective assessment format [9], [17].
- A broad range of content can be assessed on one test [5].
- Proportionally low costs [18]
- Positive cost-benefit ratio
- An existing question pool can be kept current at relatively little cost [19].

**Feasibility**

- Effort is primarily involved in generating questions; administering and grading tests require much less time and resources.
- Creating the question pool is associated with not insignificant costs [4].
- Online assessment with digital scoring is possible [5].
- A question pool shared by multiple universities increases efficiency via synergies (e.g. IMS) [20].

**Influence on teaching and learning**

- Can lead to superficial learning [21]
- Theoretical knowledge is more important than practical skills [4].
- Correct responses are already given making passive recognition possible [14].

**Structured oral examinations** (SOE) are oral assessments which are conducted by an individual examiner or a panel of examiners.

#### Structured Oral Examination

**Validity**

- Direct dependence on the degree of structuredness [22]
- Validity increases with planning, design and conditions of testing [23], [24].
- Validity is more dependent on the examiners than the method.

**Reliability**

- Increases with the number of questions, length of assessment and decreases in the face of strongly differentiated scoring [10]
- Reliability and objectivity increase with several examiners [10], [17].
- Absolute verification of reliability is practically impossible [10].
- With a Cronbach’s α of 0.65-0.88 [25], [26], [27], SOEs come out ahead of conventional exams (Cronbach’s α of 0.24-0.50) [25], [28], [29].

**Acceptance**

- Performance-inhibiting stress, anxiety and other disruptive factors play a larger role compared to MCQs [12].
- Acceptance by teachers and students is reduced by:
- Intensive supervision by examiner
- Justification of scores
- Limited information during limited time
- Questions or objections possible on the part of the student with no written test to refer to; the difference between content and type of response can lead to this [12].

**Cost**

- More cost-intensive than MCQ exams [10]
- Relativizes itself on high-stakes exams: emphasis is on reliability and validity, not on cost-effectiveness. [30], [31]

**Feasibility**

- More effort is required compared with MCQ, high financial burden resulting from need for staff and rooms / logistics [10].

**Influence on teaching and learning**

- Alongside facts, clinical reasoning, professional thinking, self-confidence and self-assurance can be assessed [12], [22].
- Since students adapt their behavior to fit a test [4], [5], [18], extensive preparation can be assumed.

If an examination is taken before a panel, the examiners consult and agree on their evaluation of the examinee’s performance. Ideally, the final grades are assigned according to a blueprint governing exam content [7].

The SOE is a testing format that enables assessment of competency level 1 (NKLZ) and beyond within the scope of usual interactions in dental care. However, the higher expenses connected with the greater need for time and personnel should be noted, as well as the potential for performance-inhibiting stress in examinees.

Use in medical and dental education

- Oral examinations with different degrees of structuredness are used in dental and medical study programs [8].
- The most important preliminary and final assessments (high-stakes exams) in both study programs (NVP, ZVP, ZP, first and second ÄP) include SOEs in various settings. Furthermore, the SOE is represented in all pre-clinical and clinical subjects in both study programs; it represents one of the traditional, predominating assessment formats [4], [32].

Assessments that do not just measure factual knowledge (=descriptive knowledge: knows) [33], [34]), but also capture the ability to apply theoretical knowledge in a specific context to solve a problem or reach a clinical decision (= procedural knowledge: knows how), require a special testing format that is indeed capable of representing this skill. It must be noted that the ability to solve problems or reason is highly specific to context and always depends on the particular context-related factual knowledge [2], [35]. In addition to the SOE, other assessment formats for evaluating procedural knowledge are the written **modified essay question** (MEQ) and key features exam. These involve case-based, written assessments that evaluate active knowledge recall, problem-solving and higher order cognitive skills while simulating clinical situations in which decisions are made in the course of a physical examination, diagnosis and therapy. A patient’s history is presented in stages, after each of which several questions are responded to in writing or by selecting the best of several possible responses. Previous questions are partially explained in the following sections making it impermissible to flip back and forth between pages.

Use in medical and dental education

- Developed in Great Britain in the 1970s for the membership examination of the Royal College of General Practitioners [36], [37], [38], [39], [40], [41].
- Used internationally in the field of medicine, from undergraduate education to post-graduate training [42], [43], [44], [45], [46], [47], [48], [49], [50].
- Used in Germany as an undergraduate testing format and as a written exam that replaces the state examination [51], [52] in model study programs (Witten/Herdecke, Cologne, Bochum, etc.).
- Hardly any examples of use in dental education; potential areas of application include assessing problem-solving skills within POL and independent learning using case-based, problem-based learning [53], practical, case-based testing with virtual patient cases (e.g. in connection with procedures for handling acute toothache in endodontics) [54].

The MEQ represents a reliable instrument to assess context-specific, procedural knowledge in clinical situations if several basic rules are adhered to: 1. inclusion of the largest number of cases possible; 2. quality control of the pre-defined grading criteria for the write-in (WI) format by several evaluators; 3. computer-based short-menu (SM) or long-menu (LM) response format. Through the simulation of decision making in a clinical setting with questions that build off of each other, learning paired with feedback becomes part of the test experience. The MEQ format represents a significant addition to the written tests commonly used at present in dental education, but it is connected with distinctly higher costs than simply running down a list of MCQs to measure purely factual knowledge.

##### Modified Essay Question

**Validity**

- Higher validity than for the MCQ format through case-based, context-rich question format [48], [55], [56]
- Contradictory results for correlation (γ) between MEQs and the results of the final exam (NBME) and post-graduate performance in the first year of professional medical practice: γ 0 0.3/0.3–0.26 [57], γ=0.51 [56].

**Reliability**

- Reliability (Cronbach’s α)=0.57–0.91 [38] depends on multiple factors [38], [39], [40], [47], [48], [58], [59]:
- Quality of the predetermined performance scale
- Response format (open-ended responses poorer than selecting from a given list)
- Number of cases and questions
- Number of graders
- → e.g. increase of Cronbach’s α from 0.7 to 0.8 by increasing the number of questions from 7 to 12 or increasing the number of graders from 1 to 4 [40].

**Acceptance**

- Students generally rate the MEQ positively [41], [51] since the MEQ format reflects practice more closely than the MCQ [60].
- Teachers/examiners: greater effort involved in creating tests, coordination challenges [51]

**Cost**

- Drafting and grading an MEQ are very time consuming and requires personnel [36], [41], [51].
- Efforts can be minimized in terms of grading by using a computer-based testing format [61].

**Feasibility**

- Generating and grading MEQs is distinctly more involved than for MCQs; difficult to design questions that actually measure the ability to solve problems or make clinical decisions and do not simply test factual knowledge [37], [41], [42], [43], [44], [45], [46], [50], [52], [53], [54], [62].

**Influence on teaching and learning**

- MEQs simulate clinical reasoning processes enabling feedback and learning during the test [39], [51], [60].

In the **key features exam** (KFE) a case unfolds in a specific clinical situation about which multiple questions are asked focusing very closely on only those critical actions or decisions (key features) that are central to the key feature problem or those that are often done incorrectly [34], [63]. Key feature cases are developed in eight defined steps [34], [64], [65]]:

1. identification of the domain or context;
2. selection of a clinical situation;
3. identification of the critical elements of the situation (key features [KF] of the problem);
4. selection and description of the clinical scenario (case vignette);
5. drafting of the questions about the key features of the problem (1-3 question per KF);
6. determination of the response format (open-ended text = write-in, selection = short menu or long menu);
7. generation of the evaluation scale; and
8. content validation.

Use in medical and dental education

- The KF assessment format proposed by Bordage und Page was developed to replace the commonly used written assessment of procedural knowledge using patient management problems (PMP) in medical specialty examinations [64], [65].
- Transfer to undergraduate education by Hatala & Norman [66], used worldwide since in medical education as a written assessment format to evaluate context-specific procedural knowledge during the study phase and post-graduate education [67], [68].
- Recognized testing format in the German-speaking countries in the field of medicine (see the detailed information on the design and implementation of assessments published by the medical schools at the Universities of Bern and Graz [34], [60], [69].
- Studies and reports on the use of the KFE as a written assessment at German medical schools, including internal medicine (Universities of Freiburg, Heidelberg, and Munich [70], Universities of Heidelberg, Tübingen [71]), hematology and oncology (University of Düsseldorf [72]), communication skills (University of Witten-Herdecke [73]).
- Extensive pilot project in veterinary medicine at the school of veterinary medicine at the University of Hanover [74].
- Only a few reports of KF problems used as a written assessment format in dental education [75], [76].

The key feature exam is a valid and reliable instrument for assessing context-specific, procedural knowledge in connection with solving a clinical problem and represents a meaningful addition to the written testing formats currently used in dental education. KFEs can also be used in independent learning with virtual patient cases. For practical reasons, the computer-based format with the long menu response format is preferable to the paper-based version. It is also easier to hinder examinees from returning to previous pages or turning the pages out of order. To increase reliability, it is better to use many short KF cases (at least 15) with a maximum of three questions each than to use fewer, more in-depth cases with four or more questions.

##### Key Features Exam

**Validity**

- High content validity (92-94%) when graded by teachers/examiners [63], [65], [67].
- Piloting and regular review of the key features by students, teachers/examiners is a pre-requisite for high content validity [34], [63], [65].
- When a LM format is intended, a WI format is recommended for the pilot to improve the quality of the LMs (supplement missing answers and distracters) [34].
- Correlation between KFE scores and other assessment scores (e.g. MCQ) is only moderate (γ=0.35-0.54, [66], [70] which can be explained by the reference to different competency levels.

**Reliability**

- Reliability of the KF format is higher than for the PMP format [65].
- Due to greater case specificity [48], reliability is directly dependent on the number of KF problems (KFP=cases) → number of cases should be as high as possible; number of questions on each case should not exceed three items, since four or more reduces reliability [77].
- The selected response format appears to influence reliability, when the same number of KF cases are used:
- 15 KFPs with 1-4 questions, 2h length, WI format: Cronbach’s α=0.49 [66]
- 15 KFPs with 3-5 questions, 1.5h length, computer-based LM format: Cronbach’s α=0.65 [70] → α=0.75 is possible with 25 KFs!

**Acceptance**

- Students: relatively high acceptance [74], [78]: evaluated as realistic and supportive of practical learning.

**Cost**

- Generation and validation of a KFE involves great amounts of time and staff [67].

**Feasibility**

- Generating KFEs is more difficult and requires more time than an MCQ [60], [69].
- Necessary testing time depends on the selected response format: LM>WI>SM>MC [79].
- The advantages of LM response format (lower cueing effect than MCQ/SM, higher inter-rater reliability than WI) can be realized by using computer-based testing with a moderate testing time [70], [72], [79].
- Testing time for 15 KFPs with 3-5 questions is 90 minutes for a computer-based exam [70] and 120 minutes for a paper-based test with a WI response format [66].
- Practical examples exist [68], [71], [75], [76], [80].

**Influence on teaching and learning**

- KFE format is closer to a real patient situation, promotes the learning of clinically relevant material and practical case-based learning [81][.

While study programs in dental medicine do impart advanced theoretical knowledge, they also require students to develop manual skills. Consequently, suitable assessment formats are needed to measure not only factual and procedural knowledge but also to give students an opportunity to demonstrate their practical abilities (shows how, [33]) and to evaluate this objectively. Simply “knows how” is raised a level to “shows how”.

When creating such assessments, the learning objectives should be selected in advance and only those which represent a practical competency level should be employed. Standardization of test and examiner allows for an objective assessment of student performance. Suitable assessment formats for this are **objective structured clinical examinations** (OSCE), objective structured practical examinations (OSPE) and the use of simulated, or standardized, patients (SP).

An OSCE is appropriate for evaluating practical skills and the ability to communicate [14]. Students pass through different stations where particular practical skills are demonstrated (including partial treatments) or mock medical consultations are conducted. Evaluations are documented using a checklist created by a group of experts according to how the exam content is weighted. Test time per station is around five minutes; two minutes need to be planned for the examinee to change stations and for the examiner to make final notes or give feedback.

Use in medical and dental education

- Widely used internationally in all clinical subjects since its introduction.
- Can be used in undergraduate and post-graduate programs [82], [83], [84], [85].
- There are many examples of use in dental disciplines: pre-clinical phase [86], [87], [88], orthodontics [89], [90], oro-maxillofacial surgery [91], [92], [93], restorative dentistry [87], [94], [95], [96], parodontology [97], clinical prosthetics [86], pediatric dentistry [98], radiology [99], microbiology [94], [97].
- Interdisciplinary OSCE [87], [94], [100], [101].
- Integration of an OSCE in the preliminary dental exam [102].
- Also used in dental education to evaluate communication skills [103], [104], problem-solving skills, and critical thinking [105].
- If possible, feedback should be included as part of the exam.

The OCSE is a reliable and valid testing format to assess individual competencies; it enjoys a high level of acceptance by students and teachers. 

##### Objective Structured Clinical Examination

**Validity**

- Predictive validity
- Significant correlation between OSCE and performance on practical tests and scores on preliminary practical medical exams p<0.01 [87]
- No correlation between OSCE and MCQ [105]
- High content and construct validities [4], [106]
- High face validity [107]
- Acceptable predictive validity [108]
- Caution required if students have a language problem or suffer from high levels of stress [109]
- Attention must be paid to the blueprint [106], [110]
- Determine content areas early [110]
- Define questions within the content areas [110]

**Reliability**

- Cronbach’s α between 0.11-0.97 [4]
- High reliability among OSCEs, with fewer than n=10 stations it being approximately 0.56, with more than n=10 stations, 0.74 [111]
- Varying recommendations on station number:
- at least 19 [4]
- 14-18 for 5-10 minutes each [106], [112]
- Stations with an SP should be assessed for at least 15 minutes [110]
- The more examiners, the higher the values.
- [111], [113] Method of evaluation critical: high values for global assessments, combinations of global assessments and checklists are good, only checklists alone are least suitable
- Post-OSCE tests increase the reliability [110].

**Acceptance**

- Students: high acceptance, appropriate testing format for functional skills [96]
- Teachers/examiners: high acceptance [112], [114], [115], [116]

**Cost**

- 181 Euros/examinee
- 86-130 Euros/examinee [4], [117]
- 2.5h/examinee [118]
- $15-200/examinee [117], [119], [120]
- Higher costs when including SPs [121]
- $21-200/examinee [122]

**Feasibility**

- Testing format demands great amounts of time and resources [106], [119]
- Thorough preparation needed:
- Establishing shared structures helps on interdisciplinary OSCEs [100].
- Evaluation by external examiners is recommended.
- Ensure the quality of SPs
- Station content should be selected to match the OSCE scenario.
- Peer reviews pre- and post-OSCE (psychometric analysis with difficulty, discrimination, etc. is recommended)
- Take the extent of the examiner’s experience, field of expertise, sex, and level of fatigue into consideration [3], [106], [112].
- Practical examples exist [95], [102].

**Influence on teaching and learning**

- Positive influence on learning [106], [108], [123]
- Stimulates learning [112]
- Learning at the stations has little to do with the reality of patients [112].
- Allot time for feedback [110]

Due to the extensive preparation involved before and after its administration, interdisciplinary cooperation is recommended to minimize this disadvantage. OSCEs can be substituted for previously used assessment formats or supplement them in meaningful ways. A sufficient number of stations (n>10), a blueprint, peer review of station content and the scoring criteria, as well as a balance among the modes of evaluation (global, checklist, combination), training the examiners and, if needed, conducting a pilot OSCE should be taken into account when designing an OSCE. A special type of OSCE is embodied in the objective structured practical examination (OSPE) during which practical skills, knowledge and/or interpretation of data are demonstrated in a non-clinical situation [124]. These assessments can be conducted in labs or simulated stations in SimLab. In contrast to the OSCE, an entire process can be evaluated through to the end result (for instance, a dental filling).

It is possible to confidently assess practical skills and/or the interpretation of clinical data with the OSPE. This format involves a reliable and valid assessment method to evaluate individual competencies; The OSPE enjoys a high level of acceptance by students and teachers. 

##### Objective Structured Practical Examination

**Validity**

- High validity, γ>7
- High construct validity

**Reliability**

- High reliability among the stations, Cronbach’s α=0.8 [125]
- Inter-rater reliability ICC>0.7
- High inter-rater reliability with equivalent levels of experience and knowledge among examiners, γ=0.79-0.93; p<0.001

**Acceptance**

- Students: high acceptance [126], [127]
- felt to be a “fair test” [128]
- preferred over traditional exam formats [126]
- Teachers: relevant, fair, objective and reliable testing format

**Cost**

- No information available

**Feasibility**

- Requires extensive planning and teamwork [128]

**Influence on teaching and learning**

- Individual competencies can be assessed, the need to demonstrate factual and procedural knowledge influences learning behavior [128].
- Makes strengths and weaknesses in practical skills discernible [129]
- Stimulates learning [129]
- Positive learning experience [130]

Defined grading criteria for each step within a process are necessary.

Use in medical and dental education

- OSPEs are administered around the world in medicine, including pharmacology [128], physiology, forensic medicine [130], and dentistry [131], [132].
- In Germany they are primarily used in the pre-clinical phase of dental education [133].

Simulated, or standardized, patients in dental education are specially trained (lay) actors who are capable of acting out common clinical pictures or typical occasions for dental consultations. They are used for both practicing and assessing doctor-patient consultations and examination techniques; the use of an SP also provides opportunities to learn how to conduct physical examinations and acquire better communication skills. It is also possible to incorporate SPs into assessments, most frequently in OSCE scenarios.

Standardized patients can be used to assess doctor-patient interactions and examination techniques. They are especially suited for evaluation of clinical competencies and communication skills within the scope of an OSCE. When implementing this, the complexity of the case should be tailored to match the testing scenario.

##### Standardized Patients

**Validity**

- Assesses clinical competencies [134]

**Reliability**

- Consistent examination
- (No significant differences between exam cohorts and time points) [135]

**Acceptance**

- Use of standardized patients (SP) within the scope of an OSCE station [136]

**Cost**

- 10-18 Euro/examinee [136]

**Feasibility**

- Case complexity can be controlled and adjusted to reflect educational level [137]
- Faculty members can determine relevant learning objectives and coordinate role creation.
- Greater need for time and staff to select and train SPs and to monitor for quality [137]
- Checklists to record all SP observations of the doctor-patient consultation [138]
- Practical examples exist [139].

**Influences on teaching and learning**

- Improves students’ clinical skills [140]

Use in medical and dental education

- This method has been used in clinical education since the 1960s [138].
- Patient contact can be simulated under standardized conditions [139].
- SPs can also provide feedback and critique the examinee’s abilities [139].

The term “workplace-based assessment” (WBA) encompasses a wide variety of testing scenarios meant to assess practical skills associated with treating patients in complex situations.

The **clinical evaluation exercise** (CEX) involves a workplace-based assessment in the clinical setting that stretches over a longer period of time (several hours to days) and covering treatment processes during which an examinee conducts a consultation with a single patient recording a patient health history and carrying out a physical examination. A maximum of two assessors should participate, but do not generally have to be present the entire time. Often the data is collected from the patient without the assessor being present. This assessment format, also known as the tCEX (traditional CEX), represents a single event measure.

Use in medical and dental education

- Originally developed in the 1960s as an assessment in internal medicine by the American Board of Internal Medicine (ABIM), it replaced the oral examination as the standard method in 1972 [141], [142].
- Replaced by the mini-CEX around 1995 [143], [144].
- No documented examples of use in dental education are found in the literature.

This assessment format is an instrument of low validity and poor reliability for testing practical skills in complex situations. It is possible to improve the assessment by using the greatest number of patients possible (cases), the greatest number of assessors possible, and the most structured evaluation instruments possible. In addition, providing feedback as part of this testing format should be mandatory. Overall, it can be asserted that in dental education the CEX is a reasonable assessment format for measuring practical competencies in complex situations only if the previously mentioned attempts at improvement have been made.

##### Clinical Evaluation Exercise

**Validity**

- Insufficient content validity; does not completely cover curricular learning objectives [145]
- Simulated situation, does not correspond with the reality of medical practice since it is too long and detailed [144]

**Reliability**

- Questionable reliability since only few exercises can be done due to the great amount of time needed [146]
- Low inter-rater reliability [147]
- Cronbach’s α is 0.24 for one case and even for two cases only 0.39 [141].

**Acceptance**

- Low level of acceptance since it is very dependent on the assessor [148]

**Cost**

- Less costly than the OSCE because real patients are used who do not need to be trained [145]

**Feasibility**

- Relatively simple since no special preparation is necessary [141]
- Practical examples exist [142], [143].

**Influence on teaching and learning**

- Patient-oriented, real-life situations [141]

The **mini-clinical evaluation exercise** (mCEX) is a patient-centered assessment format in the clinical setting that, in contrast to the CEX, requires a shorter amount of time and always includes feedback (approximately 15 minutes of assessment and 10 minutes of feedback). This testing format can be described as having three phases: observation, documentation and feedback. Over the course of the assessment, several assessors observe the examinee and evaluate what they see according to pre-defined criteria. Medical care is given to more than one patient under normal circumstances with a focus on communication and clinical examination [144]. Evaluations are generally formulated according to defined criteria valid for each examinee. These criteria can consist of a rating scale and/or short written comments. The difficulty remains in terms of the different patients undergoing physical examination. Viewed according to Miller’s pyramid, a high level of practical skill is attained. Strictly speaking, it is a structured clinical observation. 

##### Mini-Clinical Evaluation Exercise

**Validity**

- Higher validity than CEX [149]
- Acceptable validity and reliability have been demonstrated [146], [150].
- Able to validly differentiate between competency levels (first year, second year, etc.) [151]

**Reliability**

- Low inter-rater reliability [149]
- A minimum of 10 evaluations are necessary to yield reliable results; a larger number is better [151]
- At least 12-14 evaluations are recommended per year if there are different assessors to increase inter-rater reliability [152].
- Reliability of G=0.4 for 10 evaluations; G=0.8 for 50 evaluations [151]
- Dependent on number of assessors: if there is one examiner, a minimum of eight observations of different patients are necessary for a reliability of 0.8, in the case of two, four are necessary, and for three examiners, three observations [153].
- Nine items are better than five to cover differences in competencies [154].

**Acceptance**

- High level of satisfaction for students and teachers [151], [155], [156]
- Implementation is at present slow, since it involves something new [156].
- Partially problematic due to discrepancies between self-assessment and assessment by another [157].

**Cost**

- Substantial expense as a consequence of the amount of time needed [158], [159].

**Feasibility**

- Observations of authentic doctor-patient interactions by different educators in different situations; feedback on different clinical pictures at different locations each with a different focus [155]
- Thorough planning is necessary because giving feedback takes 8-17 minutes [155], [160].
- Relatively simple to implement with enough flexibility in the dental setting [161]
- Practical examples exist [162].

**Influence on teaching and learning**

- Improvement in competency through regular feedback from experts [163]
- Examiner/examinee receive feedback or a clear impression of clinical work making targeted mentoring possible [156].
- Giving constructive feedback must be learned and practiced; teaching skills are needed [164].
- No new discoveries or knowledge in comparison with traditional evaluation procedures [158]
- No influence in comparison with control groups [153]
- Learning objectives must reflect teaching content [165].
- Predictive validity between OSCE and mCEX cannot be demonstrated [165].

This assessment format is frequently referred to as the mCEX (mini-CEX) and represents a single event measure.

Use in medical and dental education

- Developed in 1995 by Norcini [144]; replaced the tCEX in the 1990s.
- Reliability depends heavily on the number of assessors and cases [151], [153].
- Several documented instances in the literature of use in dental medicine (Dental Foundation Training in Great Britain), however, often without any precise information on the evaluation instruments [161], [162].

The mCEX is a valid and reliable instrument to assess practical skills in complex situations. Options for improvement include 1. increasing the number of response items (nine are better than five) or increasing the number of observations (a minimum of 10 observations are needed) and 2. offering train-the-teacher programs (for instance in the form of video demonstrations and role playing). Longitudinal use is recommended with implementation conceivable in a wide variety of different settings (including high-stakes exams). The mCEX format is a good testing format for use in dental education to measure practical competencies in dental medicine.

Entrustable professional activities close the gap between the theory of competency-based education and patient-centered practice in a clinical context [166]. This method first became known for its use in the area of post-graduate education; since 2013 it has also appeared in undergraduate medical education [167], [168]. The integration of theoretical and practical knowledge to solve complex problems is assessed (e.g. anamnesis, clinical examination of a patient in connection with different reasons for seeking medical advice) using existing competency-based roles, such as those defined by CanMeds or ACGME. During the assessment it is determined whether the examinee is able to perform the activity while receiving directions, under supervision, with occasional assistance, or independently [169], [170]. As a result, different performance levels can be identified [171]. It is not individual learning objectives that are assessed, but rather an overall activity centering on a patient [172]. In order to differentiate EPAs from general learning objectives, it is recommended that following sentence be completed: One day, the doctor/dentist will be expected to do (insert particular activity) without direct supervision [166]. According to its definition, an EPA should include activities that are important to daily practice, very often are subject to error when being performed, and integrate multiple competencies [172], [173]. Consequently, an EPA consists of diverse roles, each role, in turn, of multiple learning objectives, and each learning objective of different performance levels. The assessment can be a direct or indirect observation and include feedback. It is crucial that the observed performance of the examinee is combined with the performance evaluation over a defined period of time. 

##### Entrustable Professional Activities

**Validity**

- High face validity [174]

**Reliability**

- Low inter-rater reliability [175]

**Acceptance**

- Potential for wide acceptance [166]
- Helps those learning to develop their own study schedule [176]
- Helps the entire faculty to maintain transparency in education [176]

**Costs**

- No information available

**Feasibility**

- Initially requires intensive, well thought-out preparation while EPAs are being designed [177]
- 20-30 EPAs are recommended for a degree program [177]
- Practical examples exist [178], [179]

**Influence on teaching and learning**

- EPAs require numerous competencies in an integrated, holistic manner [177].
- Methods of evaluation that focus on the required degree of supervision [180]
- Feedback is vital [174].
- Support from the faculty is necessary [175].
- Enables a broad (panoramic) view of the educational program [174].

A commonly reported combination is that of the mCEX with MSF (Multi-source feedback). Strictly speaking, this involves a multiple event measure.

Use in medical and dental education

- Introduced in the Netherlands by ten Cate in 2005; since then it has been used in the fields of surgery, family medicine, internal medicine, neurology, emergency medicine, pediatrics, urology, and is used widely by the Royal Australian and New Zealand College of Psychiatrists [178], [179].
- Initially in the pilot phase in German medical education [165].
- No documented instances of use in dental medicine

EPAs are a relatively new, little researched instrument for assessing practical skills in complex situations. The implementation of EPAs requires extensive and well thought-out preparation when determining the focus. To the extent possible, a maximum of 30 interdisciplinary EPAs per curricular unit should be defined drawing upon input from university instructors and practicing physicians or dentists. EPAs create a realistic link between competency-based learning objectives and higher level activities. Train-the-teacher programs (with practice giving feedback) should improve implementation. Longitudinal use is recommended. Implementation is conceivable in a wide variety of settings, including high-stakes exams. The EPA format represents an innovative approach with great future potential in terms of assessing practical skills in complex situations in dental education.

Similar to the mCEX, **Directly Observed Procedural Skills** (DOPS) entail a short workplace-based assessment in a clinical setting that includes feedback (approximately 15 minutes of assessment and 10 minutes of feedback). This also involves a three-phase assessment in which observation, documentation and feedback occur. Treatment given to (multiple) patients under conditions typical to a medical practice, as with the mCEX, but with a focus on manual skills and interventions observed by several assessors and evaluated according to defined criteria. This assessment format also represents a single event measure.

Use in medical and dental education

- Originally introduced in the United Kingdom by the General Medical Council in 2002 [144].
- Use reported in the fields of general medicine, surgery, and internal medicine [181].
- International reports of use in dentistry in Iran (universities of Shiraz and Mashad) and at Kings College in London [182], [183].

DOPS is a valid and reliable instrument to evaluate practical skills in complex situations. It is possible to improve this format by having three assessors intervene during two observations, conducting at least two observations, and by holding train-the-teacher sessions. Overall, longitudinal use is recommended. Implementation is conceivable in diverse settings, including high-stakes exams. The DOPS format is a very reasonable testing format to capture practical skills in complex situations during dental education.

##### Directly Observed Procedural Skills

**Validity**

- High face validity [181]
- Formative assessment tool [182]
- Significantly different from MCQ; provides different assessments of student performance [182]
- Separate assessment tool that does not enable an overall evaluation; a system with different possibilities is needed [184].
- DOPS efficiently evaluates practical skills [182].

**Reliability**

- To achieve a high reliability, at least three assessors should observe a student during two different case scenarios [181].
- G=0.81 [185]
- Internal consistency is 0.94 and inter-rater reliability is 0.81
- Students do not view it as suitable for improving inter-rater reliability [186].
- Substantial differences between the assessors can influence the validity of the results if there has not been strict standardization [187].
- Good reliability and consensus among assessors is possible [188].
- Fewer assessors are needed in comparison with the mCEX [160].
- Fewer assessors and cases are needed in comparison with the mCEX [181].
- Higher item correlation values than for the mCEX: 0.7-0.8 versus 0.5-0.8 [150], [189]
- Reliability depends on the case [181].
- Reliability independent of process [160]

**Acceptance**

- High acceptance by students [186]
- Examinees find the scenarios to be stressful, but appreciate the feedback [190].

**Cost**

- Substantial expense is to be expected [159], [191].

**Feasibility**

- Great amount of time needed [163], [182]
- Great amount of time needed for preparing DOPS, including giving feedback [160][
- To increase the learning effect, it is necessary to give feedback directly after the assessment and to address strengths and weaknesses [192].
- Assessors must be trained in advance [12].
- It is feasible to use only one assessor [193].

**Influence on teaching and learning**

- Examinees perceive a positive influence on independence and the learning process [186][.
- DOPS assessment improves practical clinical skills [192].
- Positive effect through directly observing the learner [192]
- Promotes an in-depth approach to learning in the clinical context [21]
- Positive influence on student reflections [181]
- Seventy percent of those observed believe that DOPS is helpful for improving practical skills [194].
- Compared to control groups there are significantly better results for DOPS regarding practical skills [195].
- Can also be used in peer arrangements in the pre-clinical and clinical context [183]

The **Portfolio **as an assessment tool is a pre-defined, objectives-centered collection of student learning activities with assigned self-reflection exercises, as well as feedback [20]. Portfolio contents are developed in alignment with the learning process; the following aspects can be taken into consideration: personal experiences (what was done, seen, written, created?), learning process (awareness that what has been experienced is relevant to future medical or dental practice), documentation (certificates, etc.), future goals regarding learning (looking ahead), and learning environments [196]. Portfolios are a multiple event measure.

Use in medical and dental education

- Portfolio-based learning was introduced in 1993 by the Royal College of General Practitioners, Portfolio assessing described by Shulman in 1998 [197], [198].
- Publications in the fields of general medicine, otorhinolaryngology, internal medicine, pediatrics, public health at universities in Maastricht (NL), Nottingham (GB), and Arkansas (USA) [196].
- Found in German medical education in Cologne [196].
- International reports of use in dentistry [199], [200], [201].

The portfolio entails a highly valid and reliable instrument for evaluating practical skills in complex situations, one that assesses collected, cumulative information about performance and development. Possibilities for optimization exist when more than one neutral grader is used, the student’s mentor is not one of these graders, and train-the-teacher sessions on giving feedback are held. Longitudinal use is recommended. Implementation is conceivable in diverse setting, including high-stakes exams. The portfolio format represents a valuable assessment format to evaluate practical skills in complex situations in dental education.

##### Portfolio

**Validity**

- Good validity if there is an appropriate selection of all required competency areas [202], [203].

**Reliability**

- Cronbach’s α is 0.8 with four graders [204]
- Cronbach’s α is 0.8 with 15 portfolio entries and two graders [202].
- Use of a clear, competency-based master plan, clear grading criteria, inclusion of guidelines and experienced graders for development and evaluation [202], [203]
- Uniform and consistent grading is difficult [200].

**Acceptance**

- Portfolios are viewed as time consuming, a source of anxiety and not very effective [205].
- The acceptance of portfolios decreases the longer students spend time on them [205].

**Cost**

- No information available

**Feasibility**

- A portfolio typically includes seven case reports, two presentations, three self-reflections [202].
- Typical content includes diagnoses and treatment plans [202].
- Problematic since there is a conflict when portfolios are used for both assessment and learning [205].
- Difficulties being self-critical and honest [205]
- Conducting interviews with students about portfolio content improved feasibility [206]
- Practical examples exist [199], [201].

**Influence on teaching and learning**

- Allows the assessment of competencies that could not otherwise be measured [200]
- Portfolio content must be aligned with the learning objectives [202].
- Increases self-knowledge and encourages critical thinking [205]
- Improves the ability to learn independently and connects theory with practice [205]
- Time consuming for grader and student [200], [207]
- Students receive constructive feedback [207].
- Calibration and validation are critically important [200].
- Provides cumulative information on performance and progress [205]
- When it is known that the portfolio will be graded, students attempt to fulfill expectations which, in turn, affects the portfolio’s content and educational value [205].
- Positive effects are heavily dependent on the support, direction, time commitment and feedback given by the teacher [205].

**Multi-source feedback**, also known as 360-degree feedback (MSF, multi-rater feedback), involves a workplace-based assessment in a clinical setting incorporating different groups of people associated with that particular work setting and the examinee (peers, dentists, nursing staff, patients, administrators, etc.). The focus of the observations is on professional conduct and teamwork, as well as the examinee taking responsibility as the person in charge [208], [209]. These aspects are observed by several assessors and evaluated according to defined criteria. The “supervisor” is given a special role in this testing scenario: this person collects all the results and gives them to the examinee. As a result, the individuals who have given feedback remain anonymous. The student receives a comprehensive picture based on all the input from different sources. High acceptance is achieved through selection of the assessors. Narrative comments and metric rating scales can be combined. This format entails a multiple event measure.

Use in medical and dental education

- Used in medicine since 1970, widespread in North America (Canada and USA), Europe (England, Holland), and Asia [210], [211].
- Reports of use in the fields of general medicine, internal medicine, surgery, gynecology, psychiatry, pathology, and radiology, etc. [210].
- Used in dental medicine by the Royal College of Surgeons of England, University of Bristol, UK Committee of Postgraduate Dental Deans.
- Validated instruments exist for evaluation (PAR: Physicians Achievement Review, SPRAT: Sheffield Peer Assessment Tool).

This method consists of a highly valid and reliable instrument for evaluating practical skills in complex situations. 

##### Multisource Evaluations

**Validity**

- Can make it easier to evaluate inter-personal and communicative skills in particular [212]
- Good validity [213]

**Reliability**

- Review: to reach a value of 0.9 minimum for Cronbach’s α, eight medical assessors, eight non-medical assessors and 25 patients must participate [210]
- High internal consistency (=0.8) with five assessors on two observed occasions [214]
- To reach a value of 0.8 for Cronbach’s α, a minimum of 11 assessors must participate [215].
- Value for Cronbach’s α is 0.98 [216].
- Problematic due to the number of assessors required [217]

**Acceptance**

- Rated 4.5 by examinees on a scale of 1-7 [214]
- Rated 5.3 by assessors on a scale of 1-7 [214]
- Evaluations are possibly too positive since anonymization is not fully trusted [217]

**Cost**

- Expense needs to be taken into account before implementation [159].

**Feasibility**

- Rated 4.4 by examinees on a scale of 1-7 [214]
- Rated 5.1 by assessors on a scale of 1-7 [214]
- Evaluations are generally verified via questionnaires making the process simple [159].
- To achieve a valid assessment, a certain number of evaluations are necessary; however, not all are possible to do [217].
- Ideally, feedback is gathered over a longer period of time [217].
- Can be easily implemented, even in a busy hospital [211], [218]

**Influence on teaching and learning**

- General improvement in clinical work, communication with co-workers and patients [219]
- Rated 4.2 by examinees on a scale of 1-7 [214]
- Rated 4.4 by assessors on a scale of 1-7 [214]
- Improvement of the evaluation process, advantage of receiving more detailed information and being exposed to different perspectives [217]
- Varying results: improvement in communication and conduct after receiving 360° feedback [220].
- Immensely time consuming and no improvement in assessment as a consequence of the feedback [221]
- It is possible to identify weak performers at an early stage [218].
- Feedback from SPs for students also possible [222].

Belonging to the success factors are a clear definition of the objectives and the sources of feedback. An important role is played by the selection of the assessors, credibility of the assessors and their familiarity with the situation under evaluation, along with the anonymity of the individuals supplying the feedback. This format can be optimized by using approximately five assessors for two observed situations and holding train-the-teacher sessions concerning constructive feedback. The combination of external feedback with self-evaluation by the examinee can be helpful, as can be jointly determining specific learning objectives for the future, including the discussion and documentation of concrete learning opportunities and supports. Longitudinal use is recommended. Implementation is also conceivable in diverse setting, including high-stakes exams. The MSF format represents a valuable assessment format for evaluating practical skills in complex situations in dental education.

## 5. Conclusion

The range of assessment methods presented in this overview significantly broadens the spectrum of already established university-specific exams—mostly MCQs and (structured) oral exams. Each of the methods outlined here meets different requirements and thus covers different competency levels. This must be taken into particular consideration by those who are involved in designing, administering and evaluating assessments in dental medicine.

When developing and implementing a curriculum, not only the choice of assessment format is critical but also noting the general functions of an exam, which in turn has an effect on the curriculum [223]: assessments can be summative or formative. Summative assessments usually come at the end of a semester or after a skill has been taught in order to evaluate learning outcomes. Formative assessments are reflective of the learning process itself and do not determine whether a student passes or fails a course or is ultimately successful in displaying the mastery of a particular competency. Such an assessment shows students their current level of proficiency and is supposed to support the learning process through reflection by students on their weaknesses. Purely formative assessments are few in the face of limited staffing resources and time constraints, but are an ideal tool for fostering the learning process.

Within the scope of drafting the NKLZ it became clear that in the future other assessment formats will be needed in addition to the established methods such as oral examinations and MC exams; these new formats will need to measure required practical skills in dental medicine, not just in the Skills Lab, but also in patient treatment. Each assessment format should correspond with the targeted competency levels.

The presentation of the assessment formats in this overview enables quick orientation within each method and makes reference to relevant literature for those who wish to know more. Including even more detailed information on each of the assessment formats would have compromised the intended character of this article as an overview. Along with theoretical knowledge of an assessment format, it is important to engage in direct exchange with colleagues in higher education who are already following a particular method. For this reason, it is desirable, and perhaps the task of the relevant working groups, to establish a network of professionals who have already gathered experience with special assessment formats and who are willing to make themselves available to those with questions. Depending upon demand, continuing education programs could emerge from such a network providing substantial assistance in implementing new assessment formats.

## 6. Outlook

With the new licensing regulations for dentists (Approbationsordnung), German dental education will be brought up to date and more closely linked to medical education. The assessment methods mentioned as examples in the NKLZ and outlined in this paper demonstrate the various options for assessing at the competency level. After experience has been gathered with university examinations in dental education and following scientific analysis of these testing methods, additional appropriate assessment methods should be included in the licensing requirements for dentistry. These should also be used to improve the quality of the state examinations.

Together with the introduction of the NKLZ, compiling experience in organizing, preparing, administering, conducting and evaluating the assessment formats profiled here will be an important task in the coming years, whereby dental medicine can make good use of the competencies under development for medical students since 2002. Dental medicine can also bring to bear its own experience and expertise in the assessment of practical skills. Our shared goal should be to continue developing assessment formats for the different competency levels in dental and medical education in cooperation with the German medical schools.

## Acknowledgements

The authors wish to extend their gratitude to all those who have helped to write, edit and finalize this article. Special thanks to the executive board of AKWLZ, especially Prof. P. Hahn, MME (University of Freiburg) and Prof. H.-J Wenz, MME (University of Kiel), for the detailed feedback and suggestions for improvement.

## Compting interests

The authors declare that they have no competing interests.

## Authors

Authors are listed in alphabetical order.

## References

[1] Biggs J (1996). Enhancing teaching through constructive alignment. High Educ.

[2] van der Vleuten CP, Verwijnen GM, Wijnen W (1996). Fifteen years of experience with progress testing in a problem-based learning curriculum. Med Teach.

[3] Norcini J, Anderson B, Bollela V, Burch V, Costa MJ, Duvivier R (2011). Criteria for good assessment: consensus statement and recommendations from the Ottawa 2010 Conference. Med Teach.

[4] Chenot JF, Ehrhardt M (2003). Objective structured clinical examination (OSCE) in der medizinischen Ausbildung: Eine Alternative zur Klausur. Z Allg Med.

[5] Examination and Assessments: Academic Integrity [Internet].

[6] Jünger J, Just I (2014). Empfehlungen der Gesellschaft für Medizinische Ausbildung und des Medizinischen Fakultätentags für fakultätsinterne Leistungsnachweise während des Studiums der Human-, Zahn-und Tiermedizin. GMS Z Med Ausbild.

[7] Nationaler Kompetenzbasierter Lernzielkatalog Zahnmedizin (NKLZ) [Internet].

[8] Möltner A, Schultz JH, Briem S, Böker T, Schellberg D, Jünger J (2005). Grundlegende testtheoretische Auswertungen medizinischer Prüfungsaufgaben und ihre Verwendung bei der Aufgabenrevision. GMS Z Med Ausbild.

[9] Norcini JJ, Swanson DB, Grosso LJ, Webster GD (1985). Reliability, validity and efficiency of multiple choice question and patient management problem item formats in assessment of clinical competence. Med Educ.

[10] Roloff S Mündliche Prüfungen [Internet].

[11] Considine J, Botti M, Thomas S (2005). Design, format, validity and reliability of multiple choice questions for use in nursing research and education. Collegian.

[12] Memon MA, Joughin GR, Memon B (2010). Oral assessment and postgraduate medical examinations: establishing conditions for validity, reliability and fairness. Adv Health Sci Educ.

[13] Harden RM, Lever R, Wilson GM (1969). Two systems of marking objective examination questions. Lancet.

[14] Harden RM, Stevenson M, Downie WW, Wilson GM (1975). Assessment of clinical competence using objective structured examination. BMJ.

[15] Lennox B (1967). Marking multiple-choice examinations. Br J Med Educ.

[16] McCarthy WH (1966). An assessment of the influence of cueing items in objective examinations. J Med Ed.

[17] Hart IR, Competence OCOAC, Harden RM, Centre RCOPASOCRSMEAR, médecins et chirurgiens du Canada des CR (1987). Further Developments in Assessing Clinical Competence.

[18] Van der Vleuten CP, Schuwirth LW, Scheele F, Driessen EW, Hodges B (2010). The assessment of professional competence: building blocks for theory development. Best Pract Res Clin Obstet Gynaecol.

[19] Schoonheim-Klein ME, Habets LL, Aartman IH, van der Vleuten CP, Hoogstraten J, van der Velden U (2006). Implementing an Objective Structured Clinical Examination (OSCE) in dental education: effects on students' learning strategies. Eur J Dent Educ.

[20] Fischer MR, Holzer M, Jünger J (2010). Prüfungen an den medizinischen Fakultäten - Qualität, Verantwortung und Perspektiven. GMS Z Med Ausbild.

[21] Cobb KA, Brown G, Jaarsma DADC, Hammond RA (2013). The educational impact of assessment: a comparison of DOPS and MCQs. Med Teach.

[22] Elmer A, Grifka J (1998). Vergleich von Prüfungsmethoden in der klinischen Ausbildung. Gesundheitswesen (Suppl Med Ausbild).

[23] Sadaf S, Khan S, Ali SK (2012). Tips for developing a valid and reliable bank of multiple choice questions (MCQs). Educ Health.

[24] Wenzel A, Kirkevang L (2004). Students'attitudes to digital radiography and measurement accuracy of two digital systems in connection with root canal treatment. Eur J Dent Educ.

[25] Yang JC, Laube DW (1983). Improvement of reliability of an oral examination by a structured evaluation instrument. J Med Educ.

[26] Hottinger U, Krebs R, Hofer R, Feller S, Bloch R (2004). Strukturierte mündliche Prüfung für die ärztliche Schlussprüfung–Entwicklung und Erprobung im Rahmen eines Pilotprojekts.

[27] Wass V, Wakeford R, Neighbour R, van der Vleuten C, Royal College of General Practitioners (2003). Achieving acceptable reliability in oral examinations: an analysis of the Royal College of General Practitioners membership examination's oral component. Med Educ.

[28] Schubert A, Tetzlaff JE, Tan M, Ryckman JV, Mascha E (1999). Consistency, inter-rater reliability, and validity of 441 consecutive mock oral examinations in anesthesiology: implications for use as a tool for assessment of residents. Anesthesiology.

[29] Kearney RA, Puchalski SA, Yang HYH, Skakun EN (2002). The inter-rater and intra-rater reliability of a new Canadian oral examination format in anesthesia is fair to good. Can J Anaesth.

[30] Postgraduate Medical Education and Training Board (2007). Developing and Maintaining an Assessment System.

[31] van der Vleuten CP Assessment of the Future [Internet].

[32] Möltner A, Schellberg D, Briem S, Böker T, Schultz JH, Jünger J (2005). Wo Cronbachs alpha nicht mehr reicht. GMS Z Med Ausbild.

[33] Miller GE (1990). The assessment of clinical skills/competence/performance. Acad Med.

[34] Kopp V, Möltner A, Fischer MR (2006). Key-Feature-Probleme zum Prüfen von prozeduralem Wissen: Ein Praxisleitfaden. GMS Z Med Ausbild.

[35] Wass V, van der Vleuten C, Shatzer J, Jones R (2001). Assessment of clinical competence. Lancet.

[36] Knox J (1989). What is.… a Modified Essay Question?. Med Teach.

[37] Knox JD, Bouchier IA (1985). Communication skills teaching, learning and assessment. Med Educ.

[38] Feletti GI (1980). Reliability and validity studies on modified essay questions. J Med Educ.

[39] Rabinowitz HK, Hojat M (1989). A comparison of the modified essay question and multiple choice question formats: their relationship to clinical performance. Fam Med.

[40] Lockie C, McAleer S, Mulholland H, Neighbour R, Tombleson P (1990). Modified essay question (MEQ) paper: perestroika. Occas Pap R Coll Gen Pract.

[41] Feletti GI, Smith EK (1986). Modified essay questions: are they worth the effort?. Med Educ.

[42] van Bruggen L, Manrique-van Woudenbergh M, Spierenburg E, Vos J (2012). Preferred question types for computer-based assessment of clinical reasoning: a literature study. Perspect Med Educ.

[43] Irwin WG, Bamber JH (1982). The cognitive structure of the modified essay question. Med Educ.

[44] Weinman J (1984). A modified essay question evaluation of pre-clinical teaching of communication skills. Med Educ.

[45] Khan MU, Aljarallah BM (2011). Evaluation of Modified Essay Questions (MEQ) and Multiple Choice Questions (MCQ) as a tool for Assessing the Cognitive Skills of Undergraduate Medical Students. Int J Health Sci.

[46] Bodkha P (2012). Effectiveness of MCQ, SAQ and MEQ in assessing cognitive domain among high and low achievers. IJRRMS.

[47] Wallerstedt S, Erickson G, Wallerstedt SM (2012). Short Answer Questions or Modified Essay questions–More Than a Technical Issue. Int J Clin Med.

[48] Elstein AS (1993). Beyond multiple-choice questions and essays: the need for a new way to assess clinical competence. Acad Med.

[49] Ferguson KJ (2006). Beyond multiple-choice questions: Using case-based learning patient questions to assess clinical reasoning. Med Educ.

[50] Palmer EJ, Devitt PG (2007). Assessment of higher order cognitive skills in undergraduate education: modified essay or multiple choice questions? Research paper. BMC Med Educ.

[51] Wild D, Rützler M, Haarhaus M, Peters K (1998). Der Modified Essay Question (MEQ)-Test an der medizinischen Fakultät der Universität Witten/Herdecke. Gesundheitswesen (Suppl Med Ausbild).

[52] Peters K, Scheible CM, Rützler M (2006). MEQ – angemessen und praktikabel? Jahrestagung der Gesellschaft für Medizinische Ausbildung - GMA. http://www.egms.de/en/meetings/gma2006/06gma085.shtml.

[53] O'Neill PN (1998). Assessment of students in a problem-based learning curriculum. J Dent Educ.

[54] Geerlings G, van de Poel AC (1984). De gestructureerde open Vraag: Een Mogelijkheit tot Patientensimulatie binnen Hetonderwijs in de Endodontologie. Ned Tijdschr Tandheelkd.

[55] Van der Vleuten CP, Schuwirth LW (2005). Assessing professional competence: from methods to programmes. Med Educ.

[56] Schwartz RW, Donnelly MB, Sloan DA, Young B (1994). Knowledge gain in a problem-based surgery clerkship. Acad Med.

[57] Rabinowitz HK (1987). The modified essay question: an evaluation of its use in a family medicine clerkship. Med Educ.

[58] Stratford P, Pierce-Fenn H (1985). Modified essay question. Phys Ther.

[59] Norman GR, Smith EK, Powles AC, Rooney PJ, Henry NL, Dodd PE (1987). Factors underlying performance on written tests of knowledge. Med Educ.

[60] Bloch R, Hofer D, Krebs R, Schläppi P, Weis S, Westkämper R (1999). Kompetent prüfen. Handbuch zur Planung, Durchführung und Auswertung von Facharztprüfungen..

[61] Lim EC, Seet RC, Oh VM, Chia BL, Aw M, Quak SH, Onk BK (2007). Computer-based testing of the modified essay question: the Singapore experience. Med Teach.

[62] Palmer EJ, Devitt PG (2007). A method for creating interactive content for the iPod, and its potential use as a learning tool: Technical Advances. BMC Med Educ.

[63] Bordage G, Brailovsky C, Carretier H, Page G (1995). Content validation of key features on a national examination of clinical decision-making skills. Acad Med.

[64] Bordage G, Page G, Hart IR, Harden RM (1987). An alternative approach to PMPs: The "key features" concept. Further developments in assessing clinical competence.

[65] Page G, Bordage G, Allen T (1995). Developing key-feature problems and examinations to assess clinical decision-making skills. Acad Med.

[66] Hatala R, Norman GR (2002). Adapting the Key Features Examination for a clinical clerkship. Med Educ.

[67] Trudel JL, Bordage G, Downing SM (2008). Reliability and validity of key feature cases for the self-assessment of colon and rectal surgeons. Ann Surg.

[68] Ali SK, Bordage G (1995). Validity of key features for a family medicine pilot exam at the College of Physicians and Surgeons Pakistan. J Coll Phys Surg Pakistan.

[69] Bernhardt J, Griesbacher T, Ithaler D, Kresse A, Öttl K, Roller-Wirnsberger R, Vogl S (2012). Kürzübersicht gängiger Prüfungsformate.

[70] Fischer MR, Kopp V, Holzer M, Ruderich F, Jünger J (2005). A modified electronic key feature examination for undergraduate medical students: validation threats and opportunities. Med Teach.

[71] Nikendei C, Mennin S, Weyrich P, Kraus B (2009). Effects of a supplementary final year curriculum on students' clinical reasoning skills as assessed by key-feature examination. Med Teach.

[72] Rotthoff T, Baehring T, Dicken HD, Fahron U, Richter B, Fischer MR, Scherbaum WA (2006). Comparison between Long-Menu and Open-Ended Questions in computerized medical assessments. A randomized controlled trial. BMC Med Educ.

[73] Zupanic M, Iblher P, Töpper J, Gartmeier M, Bauer J, Prenzel M, Möller G, Hoppe-Seyler T, Karsten G, Fischer MR (2011). Key Feature-Assessment kommunikativer Leistungen: Weiterent¬wicklung und quantitative Evaluation Jahrestagung der Gesellschaft für Medizinische Ausbildung (GMA). http://dx.doi.org/10.3205/11gma024.

[74] Schaper E, Tipold A, Ehlers JP (2013). Use of key feature questions in summative assessment of veterinary medicine students. Ir Vet J.

[75] TU Dresden Studienordnung für den Studiengang Zahnmedizin vom 08.09.2011 [Internet].

[76] Gerhardt-Szep S, Hahn P (2008). Key feature - Fallerstellung (Master of Medical Education, Modul V).

[77] Norman G, Bordage G, Page G, Keane D (2006). How specific is case specificity?. Med Educ.

[78] Huwendiek S, Mennin SP, Nikendei C (2007). Medical education after the Flexner report. N Engl J Med.

[79] Schuwirth LW, van der Vleuten CP, de Kock CA, Peperkamp AG, Donkers HH (1996). Computerized case-based testing: A modern method to assess clinical decision making. Med Teach.

[80] Huwendiek S, Reichert F, Brass K, Bosse H-M, Heid J, Möltner A, Haag M, Leven FJ, Hoffmann GF, Jünger J, Tönshoff B (2007). Etablierung von fallbasiertem computerunterstütztem Prüfen mit langen Auswahllisten: Ein geeignetes Instrument zur Prüfung von Anwendungswissen. GMS Z Med Ausbild.

[81] Huwendiek S, Heid J, Möltner A, Haag M, Tönshoff B (2008). E-Learning und E-Prüfung mit virtuellen Patienten in der Medizin.

[82] Ananthakrishnan N (1993). Microteaching as a vehicle of teacher training--its advantages and disadvantages. J Postgrad Med.

[83] Arnold RC, Walmsley AD (2008). The use of the OSCE in postgraduate education. Eur J Dent Educ.

[84] Taguchi N, Ogawa T (2010). OSCEs in Japanese postgraduate clinical training Hiroshima experience 2000-2009. Eur J Dent Educ.

[85] Pugh D, Touchie C, Wood TJ, Humphrey-Murto S (2014). Progress testing: is there a role for the OSCE?. Med Educ.

[86] Curtis DA, Lind SL, Brear S, Finzen FC (2007). The correlation of student performance in preclinical and clinical prosthodontic assessments. J Dent Educ.

[87] Eberhard L, Hassel A, Bäumer A, Becker F, Beck-Mußotter J, Bömicke W, Corcodel N, Cosgarea R, Eiffler C, Giannakopoulos NN, Kraus T, Mahabadi J, Rues S, Schmitter M, Wolff D, Wege KC (2011). Analysis of quality and feasibility of an objective structured clinical examination (OSCE) in preclinical dental education. Eur J Dent Educ.

[88] Graham R, Bitzer LA, Anderson OR (2013). Reliability and Predictive Validity of a Comprehensive Preclinical OSCE in Dental Education. J Dent Educ.

[89] Fields H, Rowland M, Vig K, Huja S (2007). Objective structured clinical examination use in advanced orthodontic dental education. Am J Orthod Dentofacial Orthop.

[90] Derringer KA (2006). Undergraduate orthodontic assessment and examination in UK dental schools. Br Dent J.

[91] Macluskey M, Durham J, Balmer C, Bell A, Cowpe J, Dawson L (2011). Dental student suturing skills: a multicentre trial of a checklist-based assessment. Eur J Dent Educ.

[92] Hoefer SH, Schuebel F, Sader R, Landes C (2013). Development and implementation of an objective structured clinical examination (OSCE) in CMF-surgery for dental students. J Craniomaxillofac Surg.

[93] Landes CA, Hoefer S, Schuebel F, Ballon A, Teiler A, Tran A, Weber R, Walcher F, Sader R (2014). Long-term prospective teaching effectivity of practical skills training and a first OSCE in Cranio Maxillofacial Surgery for dental students. J Craniomaxillofac Surg.

[94] Larsen T, Jeppe-Jensen D (20081). The introduction and perception of an OSCE with an element of self- and peer-assessment. Eur J Dent Educ.

[95] Kupke J, Wicht MJ, Stützer H, Derman SH, Lichtenstein NV, Noack MJ (2012). Does the use of a visualised decision board by undergraduate students during shared decision-making enhance patients' knowledge and satisfaction? - A randomised controlled trial. Eur J Dent Educ.

[96] Hammad M, Oweis Y, Taha S, Hattar S, Madarati A, Kadim F (2013). Students' Opinions and Attitudes After Performing a Dental OSCE for the First Time: A Jordanian Experience. J Dent Educ.

[97] Mossey PA, Newton JP, Stirrups DR (2001). Scope of the OSCE in the assessment of clinical skills in dentistry. Br Dent J.

[98] Boone WJ, McWhorter AG, Seale NS (2001). Purposeful assessment techniques (PAT) applied to an OSCE-based measurement of competencies in a pediatric dentistry curriculum. J Dent Educ.

[99] Lele SM (2011). A Mini-OSCE for Formative Assessment of Diagnostic and Radiographic Skills at a Dental College in India. J Dent Educ.

[100] Schoonheim-Klein M, Walmsley AD, Habets L (2005). An implementation strategy for introducing an OSCE into a dental school. Eur J Dent Educ.

[101] Licari FW, Knight GW (2003). Developing a group practice comprehensive care education curriculum. J Dent Educ.

[102] Ratzmann A, Wiesmann U, Kordaß B (2012). Integration of an Objective Structured Clinical Examination (OSCE) into the dental preliminary exams. GMS Z Med Ausbild.

[103] Ogawa T, Taguchi N, Sasahara H (2003). Assessing communication skills for medical interviews in a postgraduate clinical training course at Hiroshima University Dental Hospital. Eur J Dent Educ.

[104] Cannick GF, Horowitz AM, Garr DR, Reed SG, Neville BW, Day TA, Woolson RF, Lackland DT (2007). Use of the OSCE to evaluate brief communication skills training for dental students. J Dent Educ.

[105] Dennehy PC, Susarla SM, Karimbux NY (2008). Relationship between dental students' performance on standardized multiple-choice examinations and OSCEs. J Dent Educ.

[106] Khan KZ, Ramachandran S, Gaunt K, Pushkar P (2013). The Objective Structured Clinical Examination (OSCE): AMEE Guide No. 81. Part I: an historical and theoretical perspective. Med Teach.

[107] Deis N, Narciß E, Rahe J, Schüttpelz-Braun K (2012). Objektive standardisierte praktische Prüfungen zur Messung von praktischen Fertigkeiten und berufsrelevanten Kompetenzen. Z Gesundheit Sport.

[108] Beard JD, Marriott J, Purdie H, Crossley J (2011). Assessing the surgical skills of trainees in the operating theatre: a prospective observational study of the methodology. Health Technol Assess.

[109] Brand HS, Schoonheim-Klein M (2009). Is the OSCE more stressful? Examination anxiety and its consequences in different assessment methods in dental education. Eur J Dent Educ.

[110] Nikendei C, Jünger J (2006). OSCE-praktische Tipps zur Implementierung einer klinisch-praktischen Prüfung. GMS Z Med Ausbild.

[111] Brannick MT, Erol-Korkmaz HT, Prewett M (2011). A systematic review of the reliability of objective structured clinical examination scores. Med Educ.

[112] Schoonheim-Klein M, Muijtjens A, Muijtens A, Habets L, Manogue M, van der Vleuten C, Hoogstraten J, Van der Velden U (2008). On the reliability of a dental OSCE, using SEM: effect of different days. Eur J Dent Educ.

[113] Norcini JJ, Maihoff NA, Day SC, Benson JA (1989). Trends in medical knowledge as assessed by the certifying examination in internal medicine. JAMA.

[114] Hofer M, Jansen M, Soboll S (2006). Potential improvements in medical education as retrospectively evaluated by candidates for specialist examinations. Dtsch med Wochenschr.

[115] Fischer MR, Gesellschaft für Medizinische Ausbildung, Kompetenzzentrum Prüfungen Baden-Württemberg (2008). Leitlinie für Fakultäts-interne Leistungsnachweise während des Medizinstudiums: Ein Positionspapier des GMA-Ausschusses Prüfungen und des Kompetenzzentrums Prüfungen Baden-Württemberg. GMS Z Med Ausbild.

[116] Davenport ES, Davis JE, Cushing AM, Holsgrove GJ (1998). An innovation in the assessment of future dentists. Br Dent J.

[117] Rau T, Fegert J, Liebhardt H (2011). How high are the personnel costs for OSCE? A financial report on management aspects. GMS Z Med Ausbild.

[118] Kropmans TJ, O'Donovan BG, Cunningham D, Murphy AW, Flaherty G, Nestel D, Dunne FPl (2011;28). An Online Management Information System for Objective Structured Clinical Examinations. CIS.

[119] Barman A (2005). Critiques on the Objective Structured Clinical Examination. Ann Acad Med Singap.

[120] Stillman PL, Swanson DB, Smee S, Stillman AE, Ebert TH, Emmel VS, Gaslowitz J, Green HL, Hamolsky M, Hatem C (1986). Assessing clinical skills of residents with standardized patients. Ann Intern Med.

[121] Turner JL, Dankoski ME (2008). Objective structured clinical exams: a critical review. Fam Med.

[122] Carpenter JL (1995). Cost analysis of objective structured clinical examinations. Acad Med.

[123] Duerson MC, Romrell LJ, Stevens CB (2000). Impacting faculty teaching and student performance: nine years' experience with the Objective Structured Clinical Examination. Teach Learn Med.

[124] Harden RM, Cairncross RG (1980). Self assessment. Med Teach.

[125] Kundu D, Das HN, Sen G, Osta M, Mandal T, Gautam D (2013). Objective structured practical examination in biochemistry: An experience in Medical College, Kolkata. J Nat Sci Biol Med.

[126] Abraham RR, Raghavendra R, Surekha K, Asha K (2009). A trial of the objective structured practical examination in physiology at Melaka Manipal Medical College, India. Adv Physiol Educ.

[127] Adome RO, Kitutu F (2008). Creating an OSCE/OSPE in a resource-limited setting. Med Educ.

[128] Nayak V, Bairy KL, Adiga S, Shenoy S, Magazine BC, Amberkar M, Kumari MK (2014). OSPE in Pharmacology: Comparison with the conventional Method and Students' Perspective Towards OSPE. Br Biomed Bull.

[129] Wani P, Dalvi V (2013). Objective Structured Practical Examination vs Traditional Clinical Examination in Human Physiology: Student's perception. Int J Med Sci Public Health.

[130] Menezes RG, Nayak VC, Binu VS, Kanchan T, Rao PP, Baral P, Lobo SW (2011). Objective structured practical examination (OSPE) in Forensic Medicine: students' point of view. J Forensic Leg Med.

[131] Huth KC, Baumann M, Kollmuss M, Hickel R, Fischer MR, Paschos E (2015). Assessment of practical tasks in the Phantom course of Conservative Dentistry by pre-defined criteria: a comparison between self-assessment by students and assessment by instructors. Eur J Dent Educ.

[132] Banerjee R, Chandak A, Radke U (2014). Bringing objectivity to assessment in Preclinical Prosthodontics: The student's perspective on OSPE. JETHS.

[133] Schmitt I, Möltner A, Bärmeier J, Gärtner K, Dopfer S, Kuschel B, Kunkel F, Heidemann D, Gerhardt-Szép S (2013). Wie viele Prüfer braucht ein OSCE?.

[134] Adamo G (2003). Simulated and standardized patients in OSCEs: achievements and challenges 1992-2003. Med Teach.

[135] Colliver JA, Barrows HS, Vu NV, Verhulst SJ, Mast TA, Travis TA (1991). Test security in examinations that use standardized-patient cases at one medical school. Acad Med.

[136] Ortwein H, Fröhmel A, Burger W (2006). Einsatz von Simulationspatienten als Lehr-, Lern-und Prüfungsform. Psychother Psychosom Med Psychol.

[137] Collins J, Harden RM (1998). AMEE Medical Education Guide No. 13: real patients, simulated patients and simulators in clinical examinations. Med Teach.

[138] Barrows H (1993). An overview of the uses of standardized patients for teaching and evaluating clinical skills. Acad Med.

[139] Cleland JA, Abe K, Rethans JJ (2009). The use of simulated patients in medical education: AMEE Guide No 42. Med Teach.

[140] Hendrickx K, De Winter B, Tjalma W, Avonts D, Peeraer G, Wyndaele JJ (2009). Learning intimate examinations with simulated patients: The evaluation of medical students' performance. Med Teach.

[141] Norcini JJ (2002). The death of the long case?. BMJ.

[142] Norcini J (2001). The validity of long cases. Med Educ.

[143] Norcini JJ, Blank LL, Arnold GK, Kimball HR (1995). The mini-CEX (clinical evaluation exercise): a preliminary investigation. Ann Intern Med.

[144] Norcini JJ, Blank LL, Duffy FD, Fortna GS (2003). The mini-CEX: a method for assessing clinical skills. Ann Intern Med.

[145] Thornton S (2012). A literature review of the long case and its variants as a method of assessment. Educ Med J.

[146] Durning SJ, Cation LJ, Markert RJ, Pangaro LN (2002). Assessing the reliability and validity of the mini-clinical evaluation exercise for internal medicine residency training. Acad Med.

[147] Herbers JE, Noel GL, Cooper GS, Harvey J, Pangaro LN, Weaver MJ (1989). How accurate are faculty evaluations of clinical competence?. J Gen Intern Med.

[148] Yousuf N (2012). Mini clinical evaluation exercise: validity and feasibility evidences in literature. Educ Med J.

[149] Hill F, Kendall K, Galbraith K, Crossley J (2009). Implementing the undergraduate mini-CEX: a tailored approach at Southampton University. Med Educ.

[150] Kogan JR, Bellini LM, Shea JA (2003). Feasibility, reliability, and validity of the mini-clinical evaluation exercise (mCEX) in a medicine core clerkship. Acad Med.

[151] Alves De Lima A, Barrero C, Baratta S, Castillo Costa Y, Bortman G, Carabajales J, Conde D, Galli A, Degrange G, Van der Vleuten C (2007). Validity, reliability, feasibility and satisfaction of the Mini-Clinical Evaluation Exercise (Mini-CEX) for cardiology residency training. Med Teach.

[152] Norcini JJ (2005). The mini clinical evaluation exercise (mini-CEX). Clin Teach.

[153] Alves de Lima A (2013). Assessment of clinical competence: Reliability, Validity, Feasibility and Educational Impact of the mini-CEX.

[154] Cook DA, Beckman TJ (2009). Does scale length matter? A comparison of nine- versus five-point rating scales for the mini-CEX. Adv Health Sci Educ.

[155] Alves de Lima AE, Conde D, Aldunate L, van der Vleuten CP (2010). Teachers' experiences of the role and function of the mini clinical evaluation exercise in post-graduate training. Int J Med Educ.

[156] Berendonk C, Beyeler C, Westkämper R, Giger M (2008). Strukturiertes Feedback in der ärztlichen Weiterbildung: Mini-CEX und DOPS. Schweiz Ärztez.

[157] Eva KW, Regehr G (2005). Self-assessment in the health professions: a reformulation and research agenda. Acad Med.

[158] Brazil V, Ratcliffe L, Zhang J, Davin L (2012). Mini-CEX as a workplace-based assessment tool for interns in an emergency department--does cost outweigh value?. Med Teach.

[159] Magnier KM, Dale VH, Pead MJ (2012). Workplace-based assessment instruments in the health sciences. J Vet Med Educ.

[160] Wilkinson JR, Crossley JG, Wragg A, Mills P, Cowan G, Wade W (2008). Implementing workplace-based assessment across the medical specialties in the United Kingdom. Med Educ.

[161] Prescott-Clements L, van der Vleuten CP, Schuwirth LW, Hurst Y, Rennie JS (2008). Evidence for validity within workplace assessment: the Longitudinal Evaluation of Performance (LEP). Med Educ.

[162] Deshpande S, Chahande J (2014). Impact of computer-based treatment planning software on clinical judgment of dental students for planning prosthodontic rehabilitation. Adv Med Educ Pract.

[163] Veloski J, Boex JR, Grasberger MJ, Evans A, Wolfson DB (2006). Systematic review of the literature on assessment, feedback and physicians' clinical performance: BEME Guide No. 7. Med Teach.

[164] Holmboe ES, Yepes M, Williams F, Huot SJ (2004). Feedback and the mini clinical evaluation exercise. J Gen Intern Med.

[165] Montagne S, Rogausch A, Gemperli A, Berendonk C, Jucker-Kupper P, Beyeler C (2014). The mini-clinical evaluation exercise during medical clerkships: are learning needs and learning goals aligned?. Med Educ.

[166] Berberat PO, Harendza S, Kadmon M, Gesellschaft für Medizinische Ausbildung, GMA-Ausschuss für Weiterbildung (2013). Entrustable professional activities - visualization of competencies in postgraduate training. Position paper of the Committee on Postgraduate Medical Training of the German Society for Medical Education (GMA). GMS Z Med Ausbild.

[167] Colleges AOAM (2014). Core Entrustable Professional Activities for Entering Residency. [Internet].

[168] Ten Cate O (2014). Trusting graduates to enter residency: what does it take?. J Grad Med Educ.

[169] Ten Cate O, Snell L, Carraccio C (2010). Medical competence: the interplay between individual ability and the health care environment. Med Teach.

[170] Jones MD, Rosenberg AA, Gilhooly JT, Carraccio CL (2011). Perspective: Competencies, Outcomes, and Controversy—Linking Professional Activities to Competencies to Improve Resident Education and Practice. Acad Med.

[171] Mulder H, Ten Cate O, Daalder R, Berkvens J (2010). Building a competency-based workplace curriculum around entrustable professional activities: The case of physician assistant training. Med Teach.

[172] Chang A, Bowen JL, Buranosky RA, Frankel RM, Ghosh N, Rosenblum MJ, Thompson S, Green ML (2013). Transforming primary care training--patient-centered medical home entrustable professional activities for internal medicine residents. J Gen Intern Med.

[173] Scheele F, Teunissen P, van Luijk S, Heineman E, Fluit L, Mulder H, Meininger A, Wijnen-Meijer M, Glas G, Sluiter H, Hummel T (2008). Introducing competency-based postgraduate medical education in the Netherlands. Med Teach.

[174] Englander R, Carraccio C (2014). From theory to practice: making entrustable professional activities come to life in the context of milestones. Acad Med.

[175] Aylward M, Nixon J, Gladding S (2014). An entrustable professional activity (EPA) for handoffs as a model for EPA assessment development. Acad Med.

[176] Boyce P, Spratt C, Davies M, McEvoy P (2011). Using entrustable professional activities to guide curriculum development in Psychiatry training. BMC Med Educ.

[177] Ten Cate O (2013). Competency-based education, entrustable professional activities, and the power of language. J Grad Med Educ.

[178] Hauer KE, Kohlwes J, Cornett P, Hollander H, Ten Cate O, Ranji SR, Soni K, Iobst W, O'Sullivan P (2013). Identifying entrustable professional activities in internal medicine training. J Grad Med Educ.

[179] Shaughnessy AF, Sparks J, Cohen-Osher M, Goodell KH, Sawin GL, Gravel J (2013). Entrustable Professional Activities in Family Medicine. J Grad Med Educ.

[180] Ten Cate O, Scheele F (2007). Competency-based postgraduate training: can we bridge the gap between theory and clinical practice?. Acad Med.

[181] Naeem N (2013). Validity, reliability, feasibility, acceptability and educational impact of direct observation of procedural skills (DOPS). J Coll Physicians Surg Pak.

[182] Bazrafkan L (2009). Comparison of the Assessment of Dental Students'. J Med Ed.

[183] Tricio J, Woolford M, Thomas M, Lewis-Greene H, Georghiou L, Andiappan M, Escudier M (2014). Dental students' peer assessment: a prospective pilot study. Eur J Dent Educ.

[184] Abraham RR, Upadhya S, Torke S, Ramnarayan K (2005). Student perspectives of assessment by TEMM model in physiology. Adv Physiol Educ.

[185] Barton JR, Corbett S, van der Vleuten CP, English Bowel Cancer Screening Programme, UK Joint Advisory Group for Gastrointestinal Endoscopy (2012). The validity and reliability of a Direct Observation of Procedural Skills assessment tool: assessing colonoscopic skills of senior endoscopists. Gastrointest Endosc.

[186] Akbari M, Shamsabadi RM (2013). Direct Observation of Procedural Skills (DOPS) in Restorative Dentistry: Advantages and Disadvantages in Student's Point of View. Iran J Med Educ.

[187] Andersen RM, Davidson PL, Atchison KA, Hewlett E, Freed JR, Friedman JA (2005). Pipeline, profession, and practice program: evaluating change in dental education. J Dent Educ.

[188] Hamdy H, Prasad K, Williams R, Salih FA (2003). Reliability and validity of the direct observation clinical encounter examination (DOCEE). Med Educ.

[189] Torre DM, Simpson DE, Elnicki DM, Sebastian JL, Holmboe ES (2007). Feasibility, reliability and user satisfaction with a PDA-based mini-CEX to evaluate the clinical skills of third-year medical students. Teach Learn Med.

[190] Cohen SN, Farrant PBJ, Taibjee SM (2009). Assessing the assessments: U.K. dermatology trainees' views of the workplace assessment tools. Br J Dermatol.

[191] Center of Innovation in Professional Health Education and Research (CIPHER) (2007). Review of work-based assessment methods.

[192] Roghieh N, Fateme H, Hamid S, Hamid H (2013). The effect of formative evaluation using "direct observation of procedural skills" (DOPS) method on the extent of learning practical skills among nursing students in the ICU. Iran J Nurs Midwifery Res.

[193] Hamilton KES, Coates V, Kelly B, Boore JRP, Cundell JH, Gracey J (2007). Performance assessment in health care providers: a critical review of evidence and current practice. J Nurs Manag.

[194] Morris A, Hewitt J, Roberts CM (2006). Practical experience of using directly observed procedures, mini clinical evaluation examinations, and peer observation in pre-registration house officer (FY1) trainees. Postgrad Med J.

[195] Sh S, Pooladi A, BahramRezaie M, Farhadifar F, Khatibi R (2009). Evaluation of the Effects of Direct Observation of Procedural Skills (DOPS) on clinical externship students' learning level in obstetrics ward of kurdistan university of medical sciences. J Med Ed.

[196] Stosch C, Wichelhaus AS, Matthes J (2006). Die Portfolio-Methode: Modernes Assessment auf dem Prüfstand. GMS Z Med Ausbild.

[197] Buckley S, Coleman J, Davison I, Khan KS, Zamora J, Malick S, Moreley D, Pollard D, Ashcroft T, Popovic C, Sayers J (2009). The educational effects of portfolios on undergraduate student learning: a Best Evidence Medical Education (BEME) systematic review. BEME Guide No. 11. Med Teach.

[198] Tochel C, Haig A, Hesketh A, Cadzow A, Beggs K, Colthart I, Peacock H (2009). The effectiveness of portfolios for post-graduate assessment and education: BEME Guide No 12. Med Teach.

[199] Pocock I (2007). A new route for dental graduates. Dent Update.

[200] Kramer GA, Albino JEN, Andrieu SC, Hendricson WD, Henson L, Horn BD, Neumann LM, Young SK (2009). Dental student assessment toolbox. J Dent Educ.

[201] Gadbury-Amyot CC, McCracken MS, Woldt JL, Brennan RL (2014). Validity and reliability of portfolio assessment of student competence in two dental school populations: a four-year study. J Dent Educ.

[202] Michels NR, Driessen EW, Muijtjens AM, Van Gaal LF, Bossaert LL, de Winter BY (2009). Portfolio assessment during medical internships: How to obtain a reliable and feasible assessment procedure?. Educ Health.

[203] O'sullivan PS, Reckase MD, McClain T, Savidge MA, Clardy JA (2004). Demonstration of portfolios to assess competency of residents. Adv Health Sci Educ.

[204] Melville C, Rees M, Brookfield D, Anderson J (2004). Portfolios for assessment of paediatric specialist registrars. Med Educ.

[205] McMullan M (2006). Students' perceptions on the use of portfolios in pre-registration nursing education: a questionnaire survey. Int J Nurs Stud.

[206] Burch VC, Seggie JL (2008). Use of a structured interview to assess portfolio-based learning. Med Educ.

[207] Kadagad P, Kotrashetti SM (2013). Portfolio: a comprehensive method of assessment for postgraduates in oral and maxillofacial surgery. J Maxillofac Oral Surg.

[208] Brett JF, Atwater LE (2001). 360 degree feedback: accuracy, reactions, and perceptions of usefulness. J Appl Psychol.

[209] Lepsinger R, Lucia AD (2009). The Art and Science of 360 Degree Feedback.

[210] Donnon T, Al Ansari A, Al Alawi S, Violato C (2014). The reliability, validity, and feasibility of multisource feedback physician assessment: a systematic review. Acad Med.

[211] Zhao Y, Zhang X, Chang Q, Sun B (2013). Psychometric characteristics of the 360° feedback scales in professionalism and interpersonal and communication skills assessment of surgery residents in China. J Surg Educ.

[212] Joshi R, Ling FW, Jaeger J (2004). Assessment of a 360-degree instrument to evaluate residents' competency in interpersonal and communication skills. Acad Med.

[213] Archer JC, Norcini J, Davies HA (2005). Use of SPRAT for peer review of paediatricians in training. BMJ.

[214] Murphy DJ, Bruce DA, Mercer SW, Eva KW (2009). The reliability of workplace-based assessment in postgraduate medical education and training: a national evaluation in general practice in the United Kingdom. Adv Health Sci Educ.

[215] Wenrich MD, Carline JD, Giles LM, Ramsey PG (1993). Ratings of the performances of practicing internists by hospital-based registered nurses. Acad Med.

[216] Violato C, Lockyer JM, Fidler H (2008). Assessment of psychiatrists in practice through multisource feedback. Can J Psychiatry.

[217] Chandler N, Henderson G, Park B, Byerley J, Brown WD, Steiner MJ (2010). Use of a 360-degree evaluation in the outpatient setting: the usefulness of nurse, faculty, patient/family, and resident self-evaluation. J Grad Med Educ.

[218] Hesketh EA, Anderson F, Bagnall GM, Driver CP, Johnston DA, Marshall D, Needham G, Orr G, Walker K (2005). Using a 360 degrees diagnostic screening tool to provide an evidence trail of junior doctor performance throughout their first postgraduate year. Med Teach.

[219] Ferguson J, Wakeling J, Bowie P (2014). Factors influencing the effectiveness of multisource feedback in improving the professional practice of medical doctors: a systematic review. BMC Med Educ.

[220] Brinkman WB, Geraghty SR, Lanphear BP, Khoury JC, Gonzalez del Rey JA, Dewitt TG, Britto MT (2007). Effect of multisource feedback on resident communication skills and professionalism: a randomized controlled trial. Arch Pediatr Adolesc Med.

[221] Weigelt JA, Brasel KJ, Bragg D, Simpson D (2004). The 360-degree evaluation: increased work with little return?. Current Surgery.

[222] Garry A, Stirling K (2012). Achieving 360° student feedback using SPaCE. Clin Teach.

[223] Fabry G (2008). Medizindidaktik: ein Handbuch für die Praxis.

